# Global phosphotyrosine survey in triple-negative breast cancer reveals activation of multiple tyrosine kinase signaling pathways

**DOI:** 10.18632/oncotarget.5020

**Published:** 2015-09-03

**Authors:** Xinyan Wu, Muhammad Saddiq Zahari, Binyun Ma, Ren Liu, Santosh Renuse, Nandini A. Sahasrabuddhe, Lily Chen, Raghothama Chaerkady, Min-Sik Kim, Jun Zhong, Christine Jelinek, Mustafa A. Barbhuiya, Pamela Leal-Rojas, Yi Yang, Manoj Kumar Kashyap, Arivusudar Marimuthu, Min Ling, Mary Jo Fackler, Vanessa Merino, Zhen Zhang, Cynthia A. Zahnow, Edward Gabrielson, Vered Stearns, Juan Carlos Roa, Saraswati Sukumar, Parkash S. Gill, Akhilesh Pandey

**Affiliations:** ^1^ Department of Biological Chemistry, Johns Hopkins University School of Medicine, Baltimore, USA; ^2^ McKusick-Nathans Institute of Genetic Medicine, Johns Hopkins University School of Medicine, Baltimore, USA; ^3^ Department of Oncology, Johns Hopkins University School of Medicine, Baltimore, USA; ^4^ Department of Pathology, Johns Hopkins University School of Medicine, Baltimore, USA; ^5^ Institute of Bioinformatics, International Technology Park, Bangalore, India; ^6^ Department of Medicine, University of Southern California, Los Angeles, USA; ^7^ Department of Pathology, Center of Genetic and Immunological Studies (CEGIN) and Scientific and Technological Bioresource Nucleus (BIOREN), Universidad de La Frontera, Temuco, Chile; ^8^ Advanced Center for Chronic Diseases (ACCDiS), Department of Pathology Pontificia Universidad Católica de Chile, Santiago, Chile

**Keywords:** triple negative breast cancer, protein phosphorylation, kinase, AXL, proteomics

## Abstract

Breast cancer is the most prevalent cancer in women worldwide. About 15–20% of all breast cancers are triple negative breast cancer (TNBC) and are often highly aggressive when compared to other subtypes of breast cancers. To better characterize the biology that underlies the TNBC phenotype, we profiled the phosphotyrosine proteome of a panel of twenty-six TNBC cell lines using quantitative high resolution Fourier transform mass spectrometry. A heterogeneous pattern of tyrosine kinase activation was observed based on 1,789 tyrosine-phosphorylated peptides identified from 969 proteins. One of the tyrosine kinases, AXL, was found to be activated in a majority of aggressive TNBC cell lines and was accompanied by a higher level of AXL expression. High levels of AXL expression are correlated with a significant decrease in patient survival. Treatment of cells bearing activated AXL with a humanized AXL antibody inhibited cell proliferation and migration *in vitro*, and tumor growth in mice. Overall, our global phosphoproteomic analysis provided new insights into the heterogeneity in the activation status of tyrosine kinase pathways in TNBCs. Our approach presents an effective means of identifying important novel biomarkers and targets for therapy such as AXL in TNBC.

## INTRODUCTION

Breast cancer is a heterogeneous disease, with major subtypes defined by expression of estrogen receptor (ER), progesterone receptor (PR) and HER2 receptor. Approximately 70% of breast cancers are ER and/or PR positive and these tumors are typically responsive to hormonal therapies such as selective estrogen receptor modulators (SERMs) or aromatase inhibitors. Another subset of breast cancer is characterized by HER2 receptor overexpression or amplification – this subset relies on HER2 signaling for oncogenesis. HER2 receptor-positive tumors are often effectively treated with HER2-directed agents such as trastuzumab. However, about 15–20% of all breast cancer cases, classified as triple negative breast cancers (TNBCs), lack expression of these three molecules, thereby precluding the use of endocrine or anti-HER2 targeted therapies. Furthermore, TNBCs are often highly proliferative, poorly differentiated and, as a general class, are among the more aggressive subtypes of breast cancers [1–4]. Previous studies using global gene expression analyses in breast cancer identified a subtype known as basal-like breast cancers [5]. Most basal-like breast cancers also lack expression of ER, PR and HER2, and because of the significant overlap of TNBC and basal-like classes, these terms are often used interchangeably.

Studies using next-generation sequencing have demonstrated that, in addition to the two most commonly mutated breast cancer genes, *TP53* and *PIK3CA*, a large spectrum of gene mutations are present in TNBCs [6, 7]. A recent gene expression study attempted to classify TNBCs into six distinct molecular subtypes—two basal-like subtypes, an immunomodulatory, a mesenchymal, a mesenchymal stem-like and a luminal androgen receptor subtype [8]. The study also showed that several receptor tyrosine kinases including EGFR, MET and IGF1R were transcriptionally upregulated in different subsets of TNBCs. However, clinical trials in TNBCs where growth factor signaling pathways are targeted for inhibition have produced mainly discouraging results. Treatment with monoclonal anti-EGFR antibody cetuximab, alone or in combination with cytotoxic chemotherapies, demonstrated minimal improvement in progression-free and overall survival [9, 10]. The low efficacy of anti-EGFR therapy in TNBC treatment suggests that other tyrosine kinase-mediated signaling pathways might be activated in parallel in these tumors, and thus inhibiting these other aberrantly activated kinases is likely required for effective TNBC treatment.

To identify activated kinase signaling pathways in TNBC, we used quantitative mass spectrometry-based phosphoproteomics, a powerful emerging approach to directly assess the activity of protein kinases in cancer [11–13]. Two recent studies of kinase signaling in TNBC employed label-free phosphoproteomics to profile phosphotyrosine signaling in seven and fourteen TNBC cell lines, respectively. These studies identified several phosphorylation patterns that were unique to TNBCs compared to luminal breast cancers [14, 15]. For the study presented here, we aimed to extend the phosphotyrosine proteomic profiling to a broader panel of twenty six TNBC cell lines in a global, quantitative and unbiased fashion using the stable isotope labeling by amino acids in cell culture (SILAC) spike-in approach [16]. To correlate these signaling profiles with cellular phenotype, we characterized the degree of aggressiveness of each cell line using invasion assay and soft agar colony formation assay. Here, we discovered the receptor tyrosine kinase AXL to be hyperactivated and overexpressed in a majority of the most aggressive TNBC cell lines. Finally, we employed a humanized AXL monoclonal antibody developed by our group and showed that specific inhibition of AXL could attenuate TNBC cell proliferation and cell migration *in vitro* and tumor formation in mice.

## RESULTS

### Heterogeneous phenotype of triple negative breast cancer cells

**Figure 1:**
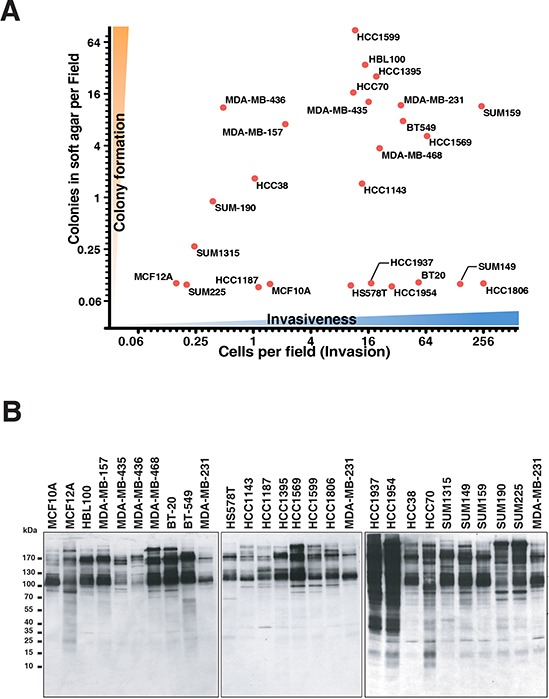
Systematic phenotyping and phosphotyrosine profiling of triple negative breast cancer cell lines **A.** Characterization of TNBC cell lines according to the extent of invasion in Boyden matrigel chamber (*x* axis) and colony formation in soft agar (*y* axis). **B.** Global protein tyrosine phosphorylation pattern across the panel of cell lines. Anti-phosphotyrosine antibody was used for immunoprecipitation and western blotting to detect tyrosine-phosphorylated proteins.

In order to systematically characterize the aggressive phenotype of all twenty-six TNBC cell lines included in our panel, we conducted two series of phenotype assays. First, soft agar colony formation assay was performed to assess anchorage-independent growth ability. Second, matrigel Boyden chamber assay was employed to assess cellular invasiveness. Systematic characterization of a large panel of TNBC cell lines using standardized conditions, which had never been performed prior to this study, enabled us to make accurate comparison between the cell lines. Even though TNBC cells are generally regarded as highly aggressive, we found that the cell lines exhibited marked variability of aggressiveness. The results from these two assays are depicted in a 2D plot in Figure 1A. In particular, we observed that ten cell lines, including MDA-MB-231, HCC1395, SUM159, HCC1599 and HCC70, demonstrated greater invasiveness and anchorage-independent growth, clustering in the upper right part of the plot. Seven cell lines, including SUM190, SUM1315, HCC38, SUM225 and HCC1187, along with two immortalized non-tumorigenic mammary epithelial cell lines MCF10A and MCF12A, clustered to the lower left part of the plot indicating low cellular invasiveness and colony forming ability. Some TNBC cell lines in our panel exhibited only high invasive ability (e.g. SUM149, HCC1806 and BT20) while others showed only high colony forming ability (e.g. MDA-MB-436 and MDA-MB157). Perhaps a surprising observation is that HBL100, an immortalized non-tumorigenic mammary epithelial cell line, exhibited an aggressive phenotype in these assays. It is likely that the observed aggressiveness, which has also been reported in other studies, is mediated by the expression of the SV40 large T antigen, which is harbored in the genome of this cell line [17–19]. The oncogenic role of SV40 was demonstrated in a study where microinjection of the SV40 DNA into normal mammary epithelial-derived cell lines was shown to confer anchorage independence and tumorigenic growth [20]. The heterogeneity that we observed in the degree of aggressive phenotype exhibited across the cell lines could be attributed to a number of different factors, including cellular origin and/or genetic lesions. Our findings thus demonstrate that there is substantial variability in cellular phenotype across TNBCs, necessitating the systematic characterization of individual cell line as performed here.

### Phosphotyrosine profiling by quantitative mass spectrometry

To assess the relative basal phosphotyrosine profiles of our panel of cell lines, we performed immunoprecipitation followed by western blot using the antiphosphotyrosine antibody 4G10. As depicted in Figure 1B, two of the non-tumorigenic breast epithelial cell lines, MCF10A and MCF12A, exhibited relatively low levels of tyrosine phosphorylation, but more than half of the tumorigenic cell lines showed strong tyrosine phosphorylation signals, with HCC1937 and HCC1954 showing the highest tyrosine phosphorylation levels. Interestingly, not all aggressive cell lines had correspondingly high levels of tyrosine phosphorylation (e.g. MDA-MB-231, HCC1599 and SUM159). Thus, abundance of tyrosine phosphorylated protein in itself is not a marker of aggressive phenotype, suggesting that the functional output of specific activated kinases may have greater significance.

**Figure 2:**
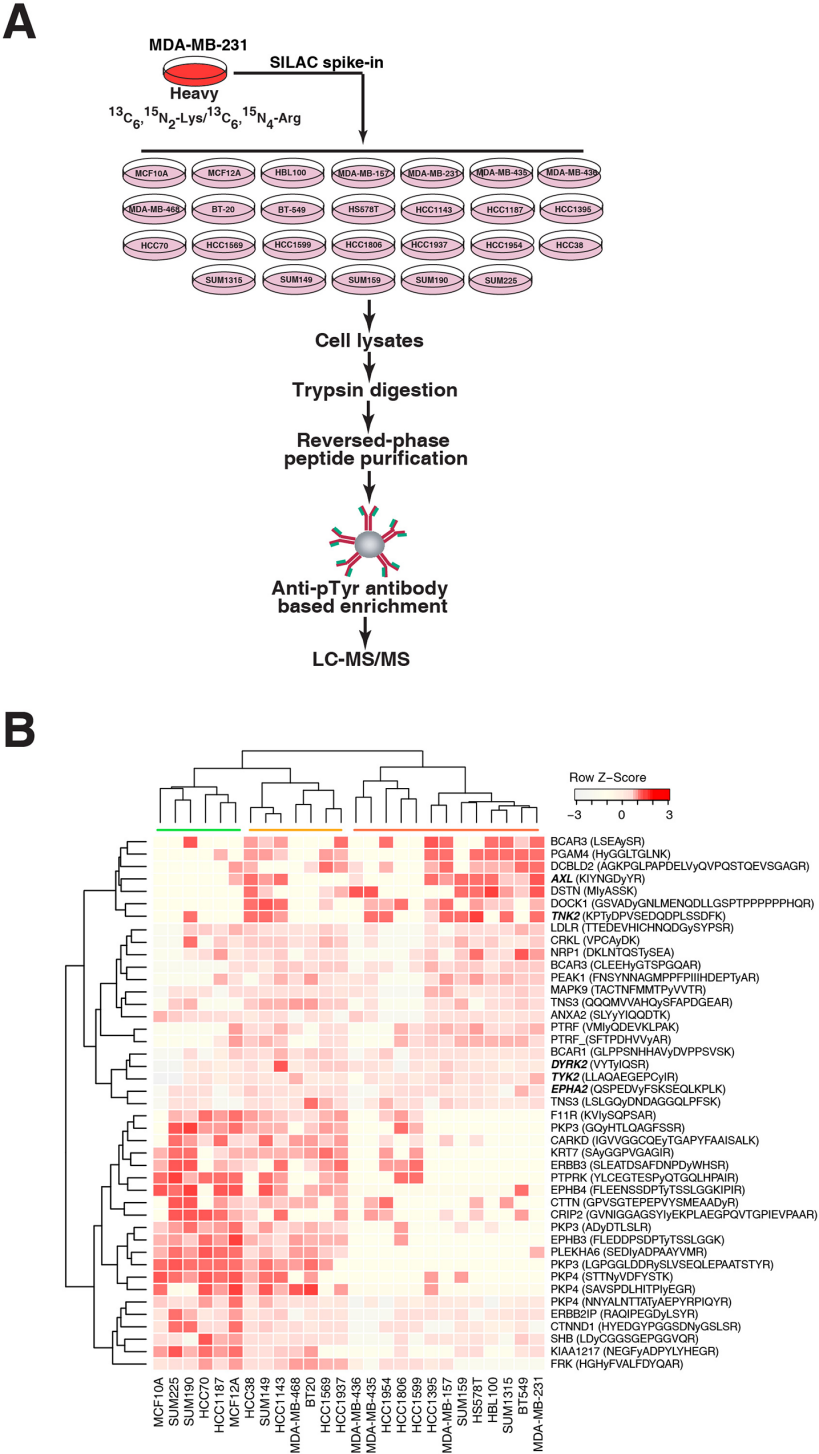
Mass spectrometry-based quantitative phosphotyrosine profiling **A.** A schematic illustration of SILAC spike-in based quantitative phosphotyrosine profiling approach. Lysates extracted from each TNBC cell line cultured in “light” medium were spiked-in with “heavy” SILAC labeled MDA-MB-231 cell lysates. The lysates were digested with trypsin followed by enrichment of phosphorylated tyrosine-containing peptides using anti-phosphotyrosine antibody (pY100). Enriched phosphotyrosine peptides were then analyzed by LC-MS/MS. **B.** Hierarchical clustering of phosphotyrosine peptides showing association with aggressive phenotypes (invasion and soft agar colony formation) of TNBC cell lines. Gene symbols along with the corresponding phosphopeptide sequences are shown. The phosphorylated tyrosine residue is indicated by a small “y”.

We next sought to identify differentially activated tyrosine kinase pathways across the TNBC cell lines using mass spectrometry-based phosphoproteomics. To accurately quantify tyrosine phosphopeptide expression across the panel of cell lines, we employed a SILAC labeling spike-in approach as described previously [21]. MDA-MB-231 cells were labeled with heavy amino acids (^13^C_6_^15^N_2_-Lys and ^13^C_6_^15^N_4_-Arg) and used as a spike-in standard to facilitate normalization across the panel of cell lines, which were grown in normal media with “light” amino acids (Figure 2A). Following SILAC spike-in into the lysates and trypsin digestion, phosphotyrosine-specific antibody-based peptide immunoprecipitation was performed to enrich for tyrosine phosphorylated peptides. The phosphotyrosine proteome of each cell line was then analyzed using a bottom-up data dependent high-resolution mass spectrometry-based approach.

In all, we identified 1,789 unique tyrosine-phosphorylated peptides (corresponding to 969 proteins) within the TNBC panel (Supplementary Tables S1 and S2). To determine the activated tyrosine kinase signaling in TNBC cells, we first quantified the phosphorylation levels of kinases across individual TNBC cell lines. Of the 54 tyrosine kinases identified from our mass spectrometry-based experiments (accounting for ∼55% of the tyrosine kinases in humans), 32 were receptor tyrosine kinases and 22 were non-receptor tyrosine kinases (Supplementary Table S2). In agreement with previous reports showing high expression of EGFR [22, 23] and MET [24, 25] in TNBCs, we detected EGFR and MET phosphorylation in almost all of the cell lines (23 and 24 cell lines, respectively). Peptides phosphorylated at kinase autophosphorylation sites were identified for a number of receptor tyrosine kinases, including EGFR, PDGFRA, FGFR1 and MET, and several non-receptor tyrosine kinase including JAK3, FER and TNK2, suggesting that these tyrosine kinases were activated and may play an important role in regulating the oncogenicity of these TNBC cells. Peptide sequences and protein identifications are detailed in Supplementary Table S2.

### Heterogeneity of TNBC revealed by phosphotyrosine profiling

We next sought to correlate the aggressive phenotypes as measured earlier with the tyrosine phosphorylation data generated by our mass spectrometry analysis. Based on our phenotype analyses, two groups of cell lines with opposing phenotypes were selected for comparison: MCF10A, MCF12A, HCC1187 and SUM225 were selected to represent non-aggressive cell lines while MDA-MB-231, HCC1569, HCC1395, BT549 and SUM159 were selected to represent highly aggressive cell lines. Here, we identified 43 tyrosine phosphorylated peptides to be differentially phosphorylated between the aggressive and non-aggressive groups (Student's *t*-test, *p* value < 0.1) (Supplementary Table S3). Using this set of peptides, we then performed clustering analysis on our entire panel of cell lines. Here, we could classify the cell lines into three clusters (Figure 2B). The right-side cluster, marked in Figure 2B by the red bar, contains cell lines with high invasiveness and strong colony forming ability. The cluster marked by the green bar represents the non-aggressive group, in which most of the cell lines are not invasive or unable to form colonies in soft agar. The cluster marked in Figure 2B by the orange bar contains cell lines that are less invasive and less likely to form colonies compared to cells in the aggressive group.

The results from the clustering analysis revealed two major patterns of peptide phosphorylation (Figure 2B); peptides associated with the top cluster exhibited relatively higher phosphorylation levels in the more aggressive TNBC cells, whereas peptides associated with the bottom cluster had relatively higher phosphorylation levels in less aggressive TNBC cells. For example, the AXL, TNK2, TYK2 and EPHA2 kinases and BCAR3, NRP1 and DSTN proteins were more phosphorylated in the more aggressive cell lines, whereas the EPHB4 kinase and PKP3, PLEKHA6, and F11R proteins, were more phosphorylated in less aggressive cell lines. These results suggest that specific tyrosine kinase signaling pathways have different biological outputs and are activated in different TNBC cells.

### Tyrosine kinases as critical mediators of the aggressive phenotypes in TNBCs

Among the hyperphosphorylated proteins that associate with aggressive TNBC cells, five are tyrosine kinases: AXL, DYRK2, TYK2, EPHA2 and TNK2. AXL, a member of the TAM receptor tyrosine kinase subfamily, has been previously implicated in the pathophysiology of multiple cancers, including breast cancer. Furthermore, overexpression of AXL has been shown to promote cancer cell proliferation and invasion and correlate with poor patient prognosis [26]. Recent studies have also reported that activation of AXL is involved in the development of EGFR inhibitor resistance in breast cancer cells [27–29]. DYRK2 is a member of the dual specificity family of kinases that autophosphorylate a critical tyrosine in their activation loop, but function only as serine/threonine kinases towards external substrates [30, 31]. In previous studies, DYRK2 activity has been shown to regulate mitotic transition and apoptosis induced by DNA damage [32, 33]. TYK2 is a member of the Janus kinase (JAK) family and is important for cytokine mediated signal transduction [34]. EPHA2 is a member of the Eph receptor tyrosine kinase family. EPHA2 was found to be overexpressed in a variety of human cancers, including breast cancer, and overexpression of EPHA2 has been shown to promote cancer cell motility and invasion [35]. TNK2, a non-receptor tyrosine kinase (also referred to as ACK1), relays phosphorylation signals from receptor tyrosine kinases such as MERTK, HER2, EGFR and PDGFR, to promote cell survival [36–39]. The *TNK2* gene has been found to be amplified in primary lung, ovarian and prostate cancers and TNK2 overexpression is also associated with poor clinical outcomes [40]. A recent immunohistochemistry-based study showed that high phosphorylation levels of TNK2 are associated with poor prognosis of breast cancer patients [41]. Oncogenic *TNK2* mutations have also been reported in lung and ovarian cancers; these cancer-associated mutations could enhance TNK2 activity to promote proliferation and migration [38, 42].

**Figure 3:**
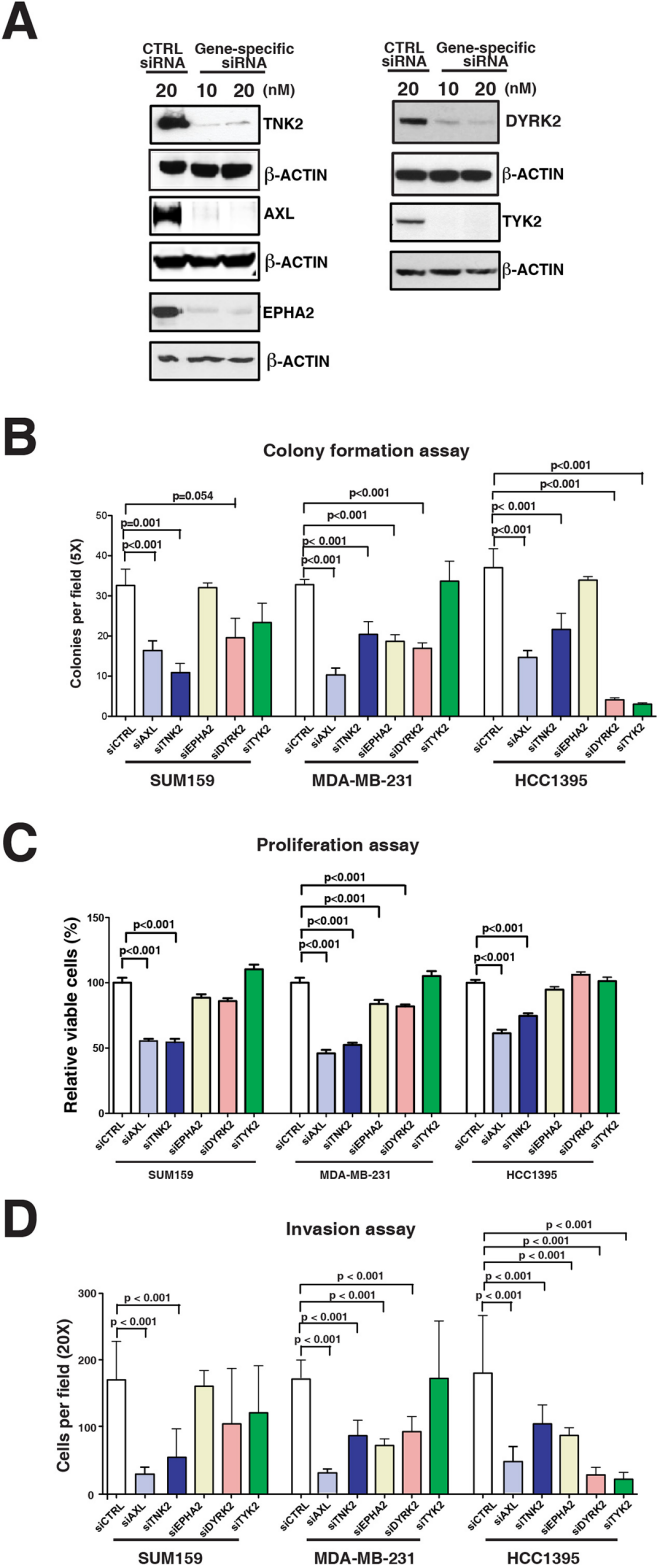
Functional validation of tyrosine kinases in aggressive phenotype of TNBC cells **A.** Western blot analysis to assess the efficiency of siRNA-based knockdown of AXL, TNK2, DYRK2, EPHA2 and TYK2 in MDA-MB-231 cells. **B–D.** The effect of siRNA-based knockdown of AXL, TNK2, DYRK2, EPHA2 and TYK2 on colony forming ability (B), proliferation (C) and invasive ability (D) of three TNBC cell lines, SUM159, MDA-MB-231 and HCC1395. Mann-Whitney tests were performed for statistical analyses.

To evaluate potential functional roles of the five tyrosine kinases identified as hyperphosphorylated in aggressive TNBC cells, we used siRNA knockdown to suppress expression of each kinase in a subset of the highly aggressive TNBC cell lines (MDA-MB-231, SUM159 and HCC1395), and proliferation, invasion and colony formation assays were then performed on each manipulated cell line (Figures 3A–3D). Inhibiting expression of AXL, DYRK2, TYK2, EPHA2 or TNK2 had variable effects on each of the assessed aggressive phenotypes for each of the TNBC cell line. For example, the colony forming ability for all three cell lines were significantly reduced with suppression of AXL and TNK2 but downregulation of TYK2 expression only affected the HCC1395 cell line (Figure 3B). Similarly, inhibiting AXL and TNK2 expression significantly diminished the proliferative ability in all three cell lines whereas EPHA2 and DYRK2 downregulation only affected the MDA-MB-231 cell line (Figure 3C). The invasive ability of all three cell lines was also significantly decreased with the knockdown of AXL and TNK2 expression, whereas the knockdown of the DYRK2 and EPHA2 affected MDA-MB-231 and HCC1395 but not SUM159 (Figure 3D). This series of experiments revealed that the knockdown of AXL and TNK2 had the most consistent effects on proliferation, invasion and colony formation across all three aggressive cell lines, suggesting that these two kinases are drivers for TNBC oncogenicity. We focused our subsequent functional studies on AXL, as it is a cell surface receptor tyrosine kinase that can be specifically targeted with a monoclonal antibody that binds, internalizes and degrades the receptor.

### AXL and phospho-AXL levels correlate with invasive phenotype in TNBC cell lines

**Figure 4:**
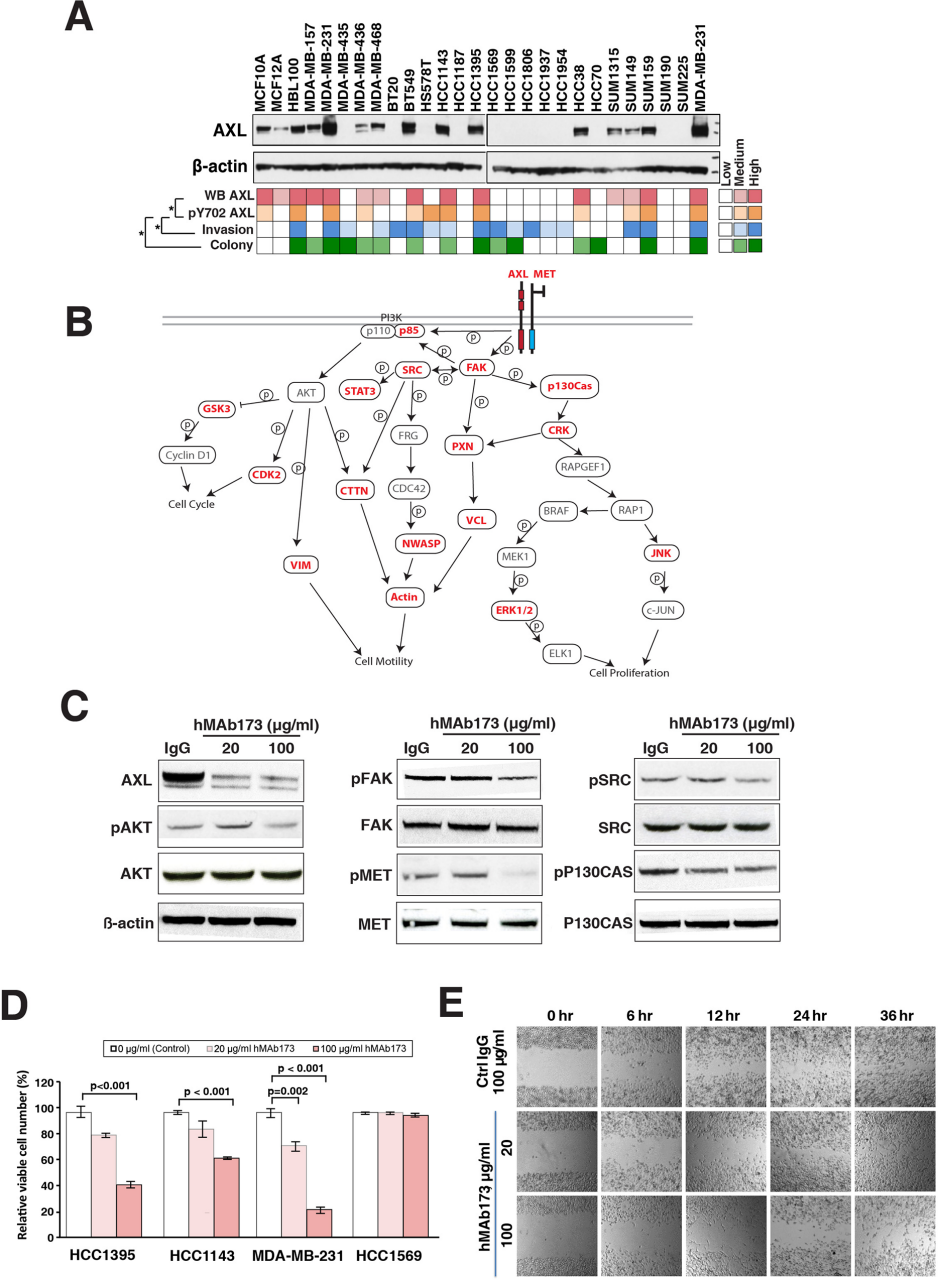
Humanized AXL antibody hMAb173 inhibits TNBC cell proliferation and migration *in vitro* **A.** AXL phosphorylation level correlates with aggressive phenotypes of TNBC cells. Top panel: Western blot analysis of the expression of AXL in the panel of TNBC cell lines. Color-coded plots showing the expression level of AXL (top row), pY702 AXL phosphorylation level (second row), invasiveness (third row) and colony formation ability (bottom row) across the panel of TNBC cells. Spearman's rank correlation was performed for statistical analysis. *p < 0.05. **B.** Activation of AXL signaling pathway in TNBC cells. Protein names in red represent the proteins commonly phosphorylated in AXL activated TNBC cells and proteins names in black represent the proteins not identified or phosphorylation not detected in all 6 AXL activated cell lines. Solid lines indicate direct phosphorylation or interaction events based on literatures and databases (KEGG, PhosphoSite). **C.** Western blot analysis to assess the AXL expression and phosphorylation levels of AXL-dowstream signaling proteins including MET (Y1003), AKT (T308), FAK (Y397), SRC (Y17) and P130Cas (Y249) in MDA-MB-231 cells treated with AXL antibody hMAb173. β-actin serves as the loading control. **D.** Cell proliferation assays of indicated cell lines treated with different doses of hMAb173 (20 μg/ml or 100 μg/ml). Mann-Whitney tests were performed for statistical analyses. **E.** Wound-healing assays to measure the cell migration ability of HCC1395 cells treated with 20 μg/ml or 100 μg/ml hMAb173. Human IgG (100 μg/ml) served as the control treatment. Microscopic observations were recorded 0, 6, 12, 24 and 36 hours after scratching the cell surface.

To obtain an overview of AXL expression levels in the panel of TNBC cell lines, we assayed for AXL protein expression by western blot. Here, we found that one-half of the twenty six TNBC panel cell lines exhibited medium to high levels of AXL expression (Figure 4A). In order to compare AXL expression levels with the levels of AXL phosphorylation identified in our phosphoproteomic profiling, we summed the ion intensities of the four unique AXL phosphopeptides with Y702 phosphorylation (IYNGDyYR, IYNGDyyR, KIYNGDyYR and KIYNGDyyR) identified in our global profiling study. We found that AXL expression levels significantly correlated with AXL phosphorylation levels (Spearman test, *p* < 0.01), indicating that AXL expression in these cells is accompanied with AXL activation. To determine if AXL phosphorylation levels correlate with aggressive phenotypes, we correlated AXL phosphorylation with anchorage independent growth and cellular invasion data as determined from the colony formation and matrigel invasion assays carried out earlier. Our analysis revealed that AXL phosphorylation levels were significantly associated with levels of anchorage-independent growth (*p* = 0.04) and cellular invasiveness (*p* = 0.01), suggesting that activation of AXL plays an important role in regulating oncogenicity of TNBC cells.

We next sought to interrogate the signaling pathways downstream of AXL in AXL-activated TNBC cell lines. Six cell lines in our panel (MDA-MB-231, BT549, HCC1143, HCC1395, HCC38 and SUM159) have the highest expression and phosphorylation level of AXL accompanied with strong colony forming and invasive ability as revealed by our phenotype assays (Figure 4A). 82 proteins from 88 phosphopeptides were found to be phosphorylated in all six cell lines (Supplementary Table S4). These 82 proteins include three receptor tyrosine kinases (EPHA2, MET and AXL) and nine downstream non-receptor tyrosine kinases including ABL1, SRC, PTK2 (also known as FAK1) and YES1. We also found a number of serine/threonine kinases, including MAPK1/3, multiple CDKs and RIPK2 to be phosphorylated in all six AXL-activated TNBC cell lines (Figure 4B and Supplementary Table S4). Many of these proteins are key signaling pathway regulators acting in concert with AXL in modulating cell proliferation and migration/invasion. These include MET, which has been shown to heterodimerize with AXL to regulate cancer cell migration/invasion [29, 43]. Major downstream effectors of AXL, including the SRC/FAK and MAPK pathways, were also found to be phosphorylated in AXL-activated cells and likely play important roles in enhancing cell proliferation and migration [44–47] (Figure 4B and Supplementary Table S4). This analysis implicated that not only AXL, but also its downstream signaling pathways, are activated in aggressive TNBC cells and hence suppressing AXL could effectively reduce the aggressiveness of TNBC cells.

### Targeting AXL with a humanized AXL antibody attenuates the tumorigenicity of TNBC cells

In order to validate the role of AXL in TNBC, we employed a humanized monoclonal antibody previously developed by our group, hMAb173 [48] to treat aggressive TNBC cells. hMAb173 specifically binds to the first fibronectin domain of human AXL and induces degradation of AXL through endocytosis [48]. As shown in Figure 4C, hMAb173 dramatically reduced AXL protein level. In order to assess the activation of AXL signaling pathway, we examined phosphorylation levels of key signaling proteins downstream of AXL that were identified in our global phosphoproteomic analysis (Figure 4B). We found that downregulating AXL expression could substantially reduce the phosphorylation levels of AKT, SRC, FAK and p130Cas (Figure 4C). This suggests that AXL expression and activation result in the activation of the PI3K-AKT and FAK-SRC signaling cascades leading to enhanced cell proliferation and migration/invasion. It has also been shown that activated AXL could form complex with SRC kinase to laterally activate the met proto-oncogene (MET) in an HGF-independent manner [43]. In our study, we also found that blockade of AXL with humanized AXL antibody could significantly decrease the phosphorylation level of MET (Figure 4C).

To study the effect of AXL suppression using hMAb173, we performed *in vitro* proliferation and wound-healing assays on MDA-MB-231, HCC1395 and HCC1143 cell lines, which are aggressive and exhibit high expression levels of AXL. The results demonstrated that hMAb173 treatment substantially suppressed cell proliferation (Figure 4D) and migration of all three cell lines (Figure 4E and Supplementary Figure S1A, S1B). As a negative control, hMAb173 treatment of HCC1569, a cell line with no detectable levels of AXL, showed no effects on cell proliferation and migration (Figure 4D and Supplementary Figure S1C).

**Figure 5:**
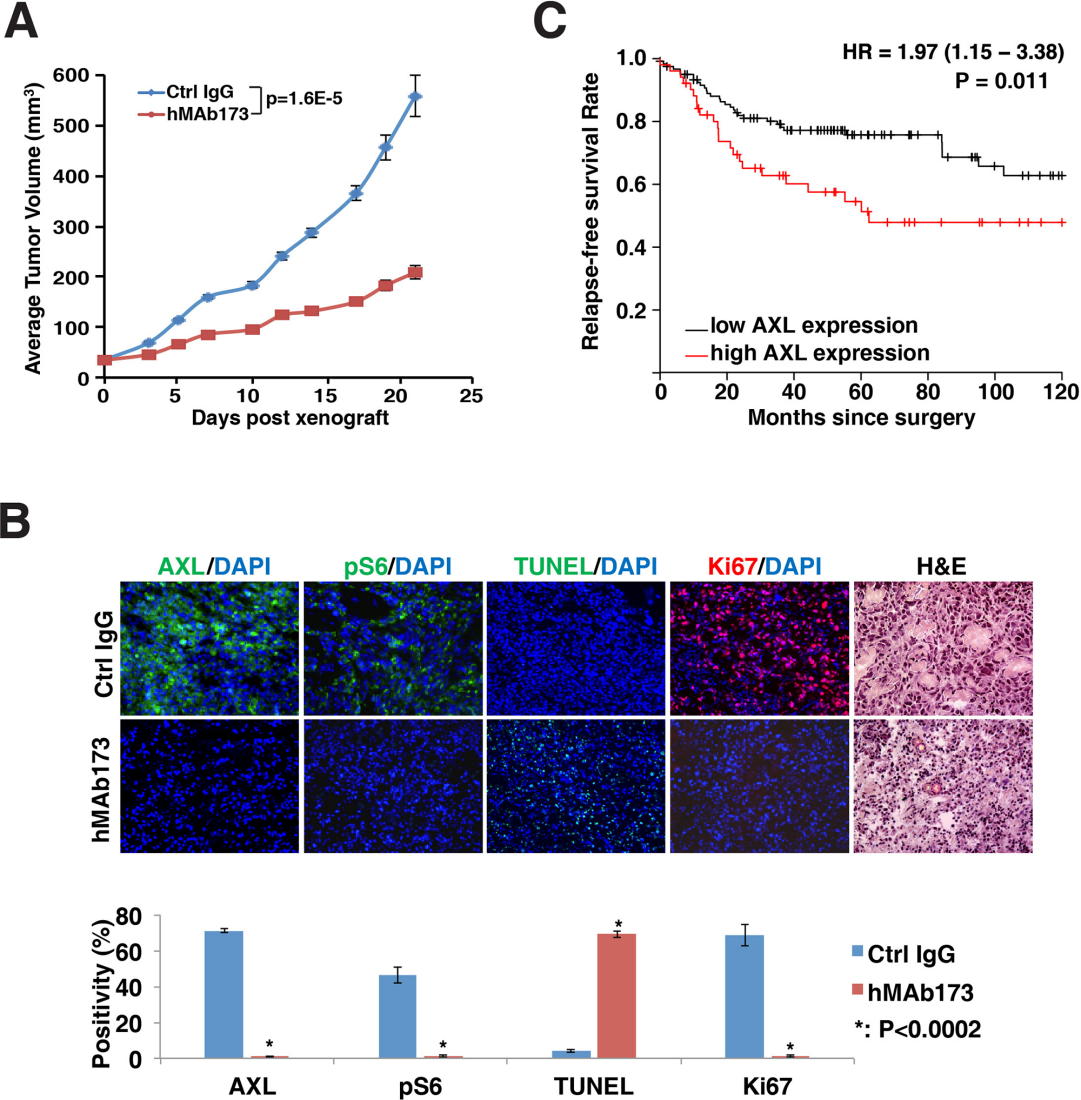
Humanized AXL antibody hMAb173 inhibits TNBC cell tumor formation *in vivo* **A.** NOD-SCID mice were implanted with MDA-MB-231 cells. When tumor sizes reached approximately 50 mm^3^, mice were treated by intraperitoneal injection of hMAb173 (20 mg/kg) or Ctrl IgG, 2 times a week. Tumor volume was measured 3 times a week and plotted. The *P* value was calculated by Student's *t* test. Mean ± SEM is shown. **B.** Top panel: Immunofluorescent staining of AXL, Ki67 and pS6 for MDA-MB-231 xenograft tumors treated with control or hMAb173 antibodies. Apoptosis was examined with TUNEL assay. Nuclei were counterstained with DAPI. Hematoxylin and eosin staining was also performed. Bottom panel: The intensity of staining and the positive signal coverage area were quantified with ImageJ (NIH) and plotted. A Student's *t*-test (two-tailed, unpaired) was used to calculate *P* values between groups where indicated. **C.** Kaplan-Meier plot of 172 high-grade ER-negative, basal-like breast cancer patients stratified with high or low AXL gene expression. The red line represents the survival curve of patients with high expression of AXL and the black line represents the survival curve of patients with low expression of AXL.

In order to assess the therapeutic potential of hMAb173, we generated TNBC tumor xenografts by transplanting MDA-MB-231 cells into immunocompromised NOD-SCID mice. Mice were then treated with 20 mg/kg hMAb173 or control IgG twice a week for three weeks. As shown in Figure 5A, the average TNBC tumor growth of the ten mice treated with hMAb173 was reduced by more than 60% compared to the control group. Immunofluorescence staining of the harvested tumors indicated that hMAb173 effectively degraded AXL in tumor cells (analyzed with a non-competing antibody), reduced tumor cell proliferation (Ki67 staining), and promoted apoptosis (TUNEL, Figure 5B). In addition, the phosphorylation of ribosomal protein S6, a major downstream effector of PI3K/AKT signaling, was significantly reduced by hMAb173 treatment (Figure 5B).

### AXL expression correlates with survival in TNBC

**Figure 6:**
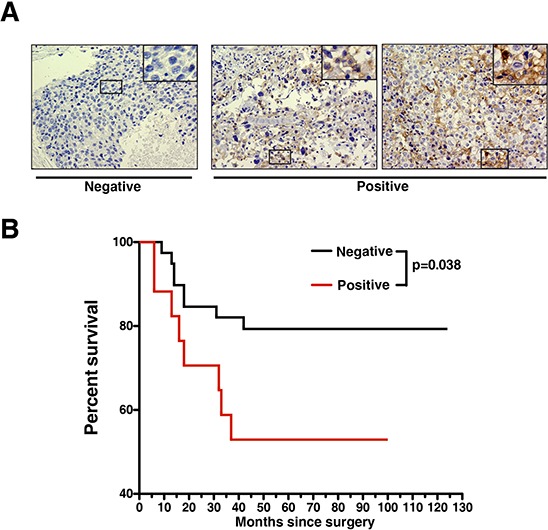
Expression of AXL protein correlates with poorer survival of TNBC patients **A.** Representative IHC staining of AXL in TNBC on breast cancer TMA indicating negative and positive staining. **B.** Kaplan-Meier plot of 57 TNBC cases stratified according to positive and negative expression of AXL, showing that AXL expression is a significant predictor of patient survival (*p* = 0.038, Mantel-Cox test).

Finally, we performed a survival analysis to examine if *AXL* gene expression correlates with clinical outcomes of breast cancer patients. Here, we utilized a publicly available gene expression database of breast tumors from 4,142 patients [49]. When we examined the data from 172 tumors that were classified as Grade 3, ER negative basal-like breast cancer, a prognostic power of AXL was observed with poorer patient outcomes (*p* = 0.01) (Figure 5C). To further evaluate AXL protein expression in TNBCs, we performed immunohistochemical (IHC) staining of AXL using a set of breast cancer tissue microarrays containing 57 TNBC tumor cores with patient survival data. We found positive AXL staining in ∼30% (17 out of 57) of TNBC cases (Supplementary Table S5). Representative AXL staining results in TNBC tumors are shown in Figure 6A. Survival analysis showed that the TNBC patient group with positive expression of AXL had significantly lower survival rate than the group with undetectable AXL expression (*p* = 0.038). This indicates that positive expression of AXL is strongly associated with poorer prognosis (Figure 6B). These data corroborate our *in vitro* and *in vivo* studies showing the important role of AXL in oncogenesis of TNBC. Taken together, these analyses demonstrate the potential of developing the humanized AXL antibody as a novel therapeutic option for TNBCs.

## DISCUSSION

In the current study, we employed a quantitative phosphoproteomic approach to quantify the intrinsic heterogeneous signaling networks present within the TNBC cell population. To do so, we applied the SILAC spike-in based metabolic labeling technique to facilitate accurate quantitation of the tyrosine phosphoproteome across a large panel of TNBC cell lines. Here, we selected twenty-six cell lines for our analysis to ensure that the heterogeneity of the TNBC subpopulation was well-represented. Two other studies have performed label-free proteomics to profile the tyrosine phosphoproteomes of seven TNBC and eight luminal breast cancer cell lines [14], and 14 TNBC and 25 luminal breast cancer cell lines [15], where it was concluded that TNBC tyrosine phosphorylation profiles differ from luminal breast cancer cell lines. In our study, we identified and quantified 1,789 unique phosphotyrosine peptides (from 969 proteins)—the largest quantitative phosphotyrosine dataset in breast cancer reported thus far. More importantly, this is the first time that the aggressive phenotype of all twenty-six TNBC cell lines has been catalogued systematically. Our data revealed that TNBC cells are not only heterogeneous at the tyrosine phosphorylation level but also at the level of oncogenic aggressiveness. Our phenotypic classification and phosphoproteome data could become a valuable resource for the breast cancer research community to help elucidate the biology of this important subpopulation of tumors.

Our phosphoproteomic analyses and functional studies specifically point to AXL as playing a major role in driving TNBC biology and aggressive phenotype. AXL has been reported to be overexpressed in a variety of cancers and to promote cancer cell proliferation, migration, invasion, and survival [26] and has drawn much attention as a potential therapeutic target in cancer. In certain cancers including breast cancer, AXL mRNA and protein levels were found to be higher in tumor metastases compared to primary tumors [50–53]. Breast cancer with epithelial-to-mesenchymal transition (EMT) phenotype predicts inferior outcome, and AXL has been shown to be an essential regulator of EMT in breast cancer [50, 54, 55]. Activation and/or overexpression of AXL has also been reported to be associated with resistance to multiple tyrosine kinase inhibitors (TKI), including erlotinib [56], lapatinib [28] and imatinib [57]. Distinct from many other receptor tyrosine kinases, *AXL* mutations are rarely found in cancer, suggesting elevated AXL expression is responsible for its oncogenic effect. Gene amplification and/or promoter hypomethylation may account for AXL overexpression [48].

Therapeutics targeting AXL are under development, including three small molecule kinase inhibitors that have entered clinical trials [58]. However, the lack of high specificity for these small molecular kinase inhibitors raises safety concerns [58]. AXL monoclonal antibodies, which are highly specific, have also been developed and reported in a few preclinical studies [59, 60]. For example, we developed a humanized antibody hMAb173 that effectively degrades AXL and inhibits proliferation/migration/invasion in AXL positive cancer cells, including TNBC cells as investigated in the current study. We also showed that hMAb173 effectively inhibits tumor growth in preclinical *in vivo* mouse models. We expect that the antibody would have even greater efficacy in an immune-competent host, where such an antibody could potentially exert antibody-dependent cell-mediated cytotoxicity and induce complement activation. Alternatively, since hMAb173 induces AXL endocytosis [48], an antibody-drug conjugate (ADC) could be designed to bring a cytotoxic drug into AXL expressing cancer cells, leading to selective cell killing. Because a majority of the aggressive subtype of TNBC has high AXL expression and phosphorylation, hMAb173 or hMAb173-ADC could have great potential clinical applications. hMAb173 may also be used for imaging TNBC tumors, and we have successfully used fluorescence- or radio-labeled hMAb173 to image a lung tumor xenograft *in vivo* [61, 62]. Similarly, hMAb173-based imaging can be used to select TNBC patients who exhibit high AXL expression for AXL-targeted therapy.

In summary, our SILAC-based quantitative phosphoproteomic approach revealed high heterogeneity of the tyrosine phosphoproteome of TNBC cell lines. Correlating the tyrosine profiles with the aggressive phenotypes and siRNA knockdown-based functional screening, we identified AXL as an important mediator of aggressiveness of TNBC cells. More importantly, we demonstrated that the humanized monoclonal antibody hMAb173 has high potential to serve as a novel therapeutic agent for the treatment of highly aggressive triple negative breast cancer.

## MATERIALS AND METHODS

### Cell culture and reagents

To construct a comprehensive panel representative of the heterogeneous nature of the TNBC subtype, 26 triple negative breast cancer cell (from the IBC45 panel gifted by NCI/ATCC) lines were cultured in appropriate growth media. MDA-MB-231 cells were also grown in heavy SILAC medium (^13^C_6_^15^N_2_-Lysine (K8) and ^13^C_6_^15^N_4_-Arginine (R10)) to serve as an internal control for normalization of phosphopeptide quantitation. For harvesting cell lysates for phosphoproteomic analysis, cells were seeded at 80% confluency two days ahead of harvest. Cells were serum starved overnight before harvesting with urea lysis buffer containing 20 mM HEPES pH 8.0, 9 M urea, 1 mM sodium orthovanadate, 2.5 mM sodium pyrophosphate, 1 mM Δ-glycerophosphate and 5 mM sodium fluoride.

### Anti-phosphotyrosine antibody immunoprecipitation, western blot and siRNA knockdown

Each cell line was harvested and lysed in modified RIPA buffer (50 mm Tris-HCl, pH 7.4, 150 mm NaCl, 1 mm EDTA, 1% Nonidet P-40, 0.25% sodium deoxycholate, and 1 mm sodium orthovanadate in the presence of protease inhibitors) and immunoprecipitation was performed as previously described [13]. Briefly, whole cell protein extracts were subjected to immunoprecipitation using agarose-conjugated 4G10 antibody in modified RIPA buffer at 4°C for 2 hours. Sample loading buffer was added and incubated at 90°C for 5 minutes. Immunoprecipitated samples were separated in NuPAGE gels (Invitrogen), transferred to nitrocellulose membranes (GE), and probed with horseradish peroxidase-conjugated 4G10 antibody. Other primary antibodies used in this study are anti-TNK2 (Santa Cruz Biotechnology), anti-DYRK2 (Cell Signaling Technology), anti-EPHA2 (Epitomics), anti-TYK2 (Cell Signaling Technology), anti-AKT (Cell Signaling Technology), anti-phospho AKT (T308) (Cell Signaling Technology), anti-AXL (Cell Signaling Technology), anti-P130Cas (Cell Signaling Technology), anti-phospho P130Cas (Y249) (Cell Signaling Technology), anti-phospho FAK (Y397) (Cell Signaling Technology), anti-FAK (Cell Signaling Technology), anti-phospho MET (Y1003) (Cell Signaling Technology), anti-MET (Cell Signaling Technology), anti-phospho SRC (Y17) (Cell Signaling Technology), and anti-SRC (Cell Signaling Technology). 50 nM siRNA targeting AXL (CAAGAUUCUAGAUGAUUAATT), EPHA2 (GGAAGUGGUACUGCUGGACTT), TNK2 (L-003102, SMARTpool, Dharmacon), DYRK2 (L-004730, SMARTpool, Dharmacon), TYK2 (L-003182, SMARTpool, Dharmacon) was used for transfections with RNAiMax (Invitrogen). Cells were harvested 48 hours post-transfection or treatment with hMAb173 for assessing knockdown efficiency, western blot analyses or other follow-up experiments.

### In-solution trypsin digestion

MDA-MB-231 cells were grown in heavy SILAC medium containing R10 and K8 for six rounds of label incorporation. Heavy labeled MDA-MB-231 cells were then harvested and lysed as per the protocols described above. To facilitate normalization applied to the quantitative analysis of the proteomics dataset, heavy labeled MDA-MB-231 cell lysate was spiked into each of the 26 prepared TNBC cell lysates at a 1:5 ratio prior to mass spectrometric experiments. 25 mg protein (20 mg from each light labeled cell line and 5 mg from heavy labeled MDA-MB-231 cells) were reduced with 5 mM dithiothreitol, alkylated with 10 mM iodoacetamide and then diluted to a final concentration of less than 2 M urea using 20 mM HEPES (pH 8.0). In-solution digestion was then carried out using TPCK-treated trypsin on an orbital shaker at 25°C overnight. The reaction was quenched using 1% trifluoroacetic acid (TFA) and the digests were cleared by centrifugation and desalted using SepPak C18 cartridge. Eluted peptides were lyophilized and subjected to phosphotyrosine peptide enrichment.

### Immunoaffinity purification of phosphotyrosine peptides

Lyophilized peptides were reconstituted in 1.4 ml of immuno-affinity purification (IP) buffer containing 50 mM MOPS pH 7.2, 10 mM sodium phosphate, 50 mM NaCl. Anti-phosphotyrosine antibody (pY100, Cell Signaling Technology) was mixed with peptide solution and incubated on a rotator at 4°C for 45 minutes. Post-incubation, the pY100 antibody and phosphotyrosine peptide complex were washed with IP buffer and water. The phosphotyrosine peptides were eluted using 0.1% TFA. The eluted peptide samples were desalted using C_18_ STAGE tips, vacuum dried and kept at −80°C before LC-MS analysis.

### Liquid chromatography tandem mass spectrometry

LC-MS/MS analysis of enriched phosphotyrosine peptides was carried out using a reversed-phase liquid chromatography system interfaced with an LTQ-Orbitrap Velos mass spectrometer (Thermo Scientific) essentially as previously described [13]. The peptides were loaded onto an enrichment column (2 cm × 75 μm, Magic C_18_ AQ 5 μm, 120 Å) with a flow rate of 3 μl/min using 0.1% formic acid in water. Peptides were separated on an analytical column (10 cm × 75 μm, Magic C_18_ AQ 5 μm, 120 Å). Precursor scans (FTMS) from 350-1,700 m/z at 60,000 resolution followed by MS2 scan (FTMS) of HCD fragmentation of the 10 most abundant ions (isolation width: 1.50 m/z; normalized collision energy: 35%; activation time = 0.1 ms, default charge state: 2) at 7,500 resolution. Running time was set to 135 minutes.

### Mass spectrometry data analysis

The tandem mass spectrometry data were searched using MASCOT (Version 2.2.0) and SEQUEST search algorithms against a Human RefSeq database (version 59, containing 33,249 protein entries) supplemented with frequently observed contaminants through the Proteome Discoverer platform (version 1.4, Thermo Scientific). For both algorithms, the search parameters included a maximum of one missed cleavage; carbamidomethylation at cysteine as a fixed modification; oxidation at methionine, phosphorylation at serine, threonine and tyrosine and SILAC labeling ^13^C_6,_^15^N_2-_-Lysine; ^13^C_6,_^15^N_4_-Arginine as variable modifications. The MS tolerance was set at 10 ppm and MS/MS tolerance to 0.05 Da. The false discovery rate was set to 0.01 at the peptide level. The quantitation ratio for each phosphopeptide-spectrum match (phosphoPSM) was calculated by the quantitation node and the probability of phosphorylation for each Ser/Thr/Tyr site on each peptide was calculated by the PhosphoRS node (Version 3.0) in the Proteome Discoverer. Phosphorylation sites were assigned based on the phosphoRS probability ≥ 75% threshold. Since phosphotyrosine peptides were specifically enriched for mass spectrometry analysis, if the phosphoRS probabilities of ambiguous sites are same for tyrosine or serine/threonine residues, we assigned phosphorylation onto the tyrosine residue.

In order to quantify peptide phosphorylation levels across the panel of cell lines, intensity measurements of all detected heavy phosphopeptides identified from SILAC heavy labeled MDA-MB-231 lysates spiked-in with each cell line lysates were summed. The ratio of summed intensity of heavy phosphopeptides from each cell line mix versus average intensity of heavy phosphopeptides across all 26 cell lines was calculated and served as normalization factor.

### Statistical analysis and hierarchical clustering analysis

Phosphopeptides identified in common in more than 15 of the 26 studied cell lines were used for statistical analysis. If the phosphopeptide was not identified in certain cell lines, we imputed the missing value with the half of the minimal intensity of the same phosphopeptide across the cell lines. To consolidate the phosphopeptides, peptides with the same sequence were merged and the intensity of these peptides were summed. The phosphopeptides with small variances were further filtered out using the cutoff as the mean of all variances across all cell lines. Finally, the data set for the subsequent statistical analyses contains 239 unique peptides across 26 cell lines.

For the supervised analysis, we first selected two groups of cell lines among 26 cell lines according to their invasiveness and the ability of colony formation, where five cell lines (HCC1395, MDA-MB-231, HCC1569, SUM159, and BT549) were selected as the aggressive group and four cell lines (MCF10A, MCF12A, SUM225, and HCC1187) were selected as non-aggressive group. Student's *t*-test was performed on these two groups to identify peptides whose phosphorylation levels were different between the two populations (*p*-value < 0.1). Hierarchical clustering analysis was then performed in R environment using heatmap.2 function.

### Matrigel invasion assays

Cells were washed once with PBS, detached using trypsin (Life Technologies) and 5 × 10^4^ cells were seeded into Biocoat matrigel invasion chambers (BD Biosciences). Growth media supplemented with serum for each cell line was added in the lower wells as the chemoattractant. After 24 hours, the filter membranes were stained with DAPI (Invitrogen). The number of cells that penetrated through the matrigel and membrane was counted for ten randomly selected viewing fields at 20x magnification.

### Migration wound-healing assay

Cancer cells were seeded into 24-well plates and cultured until confluent. A wound was created by scraping the cell monolayer with a sterile pipette tip. Cells were washed with PBS and fresh culture medium was added. Cells were then treated with control human IgG or hMAb173 for up to 36 hours. The healing process was examined dynamically with a Leica S40 microscope equipped with a Leica DFC340 FX digital camera.

### Soft agar colony formation assays

Briefly, 1.5 ml of 0.5% bottom layer agar was prepared in six-well plates. Cells with different treatments were separately trypsinized, centrifuged, resuspended in 0.35% agar medium (equal volumes of 0.7% agar and culture medium), and plated onto the top agar at 5,000 cells per well. The cells were grown for 14 days at 37°C. Colonies were then stained with crystal violet and counted under the microscope.

### MTT cell proliferation assay

MTT (3-(4, 5-dimethylthiazol-2-yl)-2, 5-diphenyltetrazolium bromide) assays were performed to measure the cell proliferation. Briefly, cells that were transfected with different siRNAs in 96-well plate were left to grow for 5–7 days before the MTT assay. 1 mg/ml MTT in growth media was added into each well and the plate was incubated for two hours in 37°C. Media was then removed and 100 μl of DMSO and ethanol (1:1 by volume) was added into each well. The signal intensity was measured at 530 nm on a microplate reader and data were presented as optical density.

### *In vivo* tumor xenograft assays

To evaluate the therapeutic potential of targeting AXL with humanized AXL antibody hMAb173, 3 × 10^6^ MDA-MB-231 cells were injected subcutaneously into the flanks of female NOD-SCID mice. Tumor growth was measured three times a week, and volume was estimated as 0.52 × length × width^2^. Once tumors were established (∼50 mm^3^), animals were distributed into treatment and control groups (*n* = 10). Each group was treated by intraperitoneal injection of 20 mg/kg of antibody 2 times a week. At the end of the experiment, mice were sacrificed for tissue analysis. All procedures were approved by USC institutional Animal Care and Use Committee and were performed in accordance with the Animal Welfare Act regulations.

### Immunofluorescence staining of xenograft tumors

Tumors were harvested and immediately snap frozen. 5-μm sections were fixed in 4% paraformaldehyde, blocked with goat serum, and incubated with rabbit antibodies against Ki67 (Abcam, Cambridge, MA), AXL, and S6 phosphorylated at Ser235/Ser236 (both from Cell Signaling, Danvers, MA) overnight at 4°C. Antibody binding was localized with appropriate AlexaFluor-conjugated secondary antibodies (Invitrogen, Carlsbad, CA). Apoptosis was analyzed using terminal deoxynucleotidyl transferase–mediated dUTP nick end labeling (TUNEL) fluorescent kit (Roche, Nutley, NJ) following the manufacturer's instructions. Nuclei were counterstained with 6-diamidino-2-phenylindole dihydrochloride hydrate (DAPI). Images were obtained with a Nikon Eclipse 80i fluorescence microscope and Meta Morph imaging series system. The intensity of staining and the positive signal coverage area were quantified with ImageJ (NIH). Student's *t*-test (two-tailed, unpaired) was used to calculate *P* values between groups where indicated.

### Immunohistochemistry

Paraffin-embedded breast cancer tissue macroarray (TMA) sections were deparaffinised in xylene, rehydrated through graded ethanol solutions, and washed in distilled water. For antigen retrieval, the sections were heated in citrate buffer (pH 6) for 20 min at 95°C and then cooled the sections for 20 minutes at room temperature. Endogenous peroxidase activity was quenched by incubating the sections in 3% H_2_O_2_ in H_2_O for 5 minutes. The TMA sections were then incubated with anti-Axl antibody (rabbit monoclonal, Cell Signaling clone C89E7; 1:300 dilution) at 4°C overnight, with anti-rabbit amplifier antibody (Vector Laboratories) at room temperature for 30 minutes and finally with ImmPress Excel polymer reagent (Vector Laboratories) at room temperature for 30 minutes. Signal was developed using ImmPACT DAB EqV Q8 working solution (Vector Laboratories) and pictures were taken with an Olympus BX51 microscope equipped with a Retiga 200R camera (Qimaging). Staining was scored in a semi-quantitative manner where positivity of > 5% of the tumor cells was necessary for scoring a case as positive. Staining intensity in positive cases was graded on a scale of 1–3.

## SUPPLEMENTARY FIGURE AND TABLES










