# Supplementary material for: Global phosphotyrosine survey in triple-negative breast cancer reveals activation of multiple tyrosine kinase signaling pathways
Source: Oncotarget. 2015 Sep 3;6(30):29143–60. doi: 10.18632/oncotarget.5020 (PMC4745717; doi:10.18632/oncotarget.5020)
Supplement: Supplementary file 1 [file oncotarget-06-29143-s001.pdf]

SUPPLEMENTARY FIGURE AND TABLES

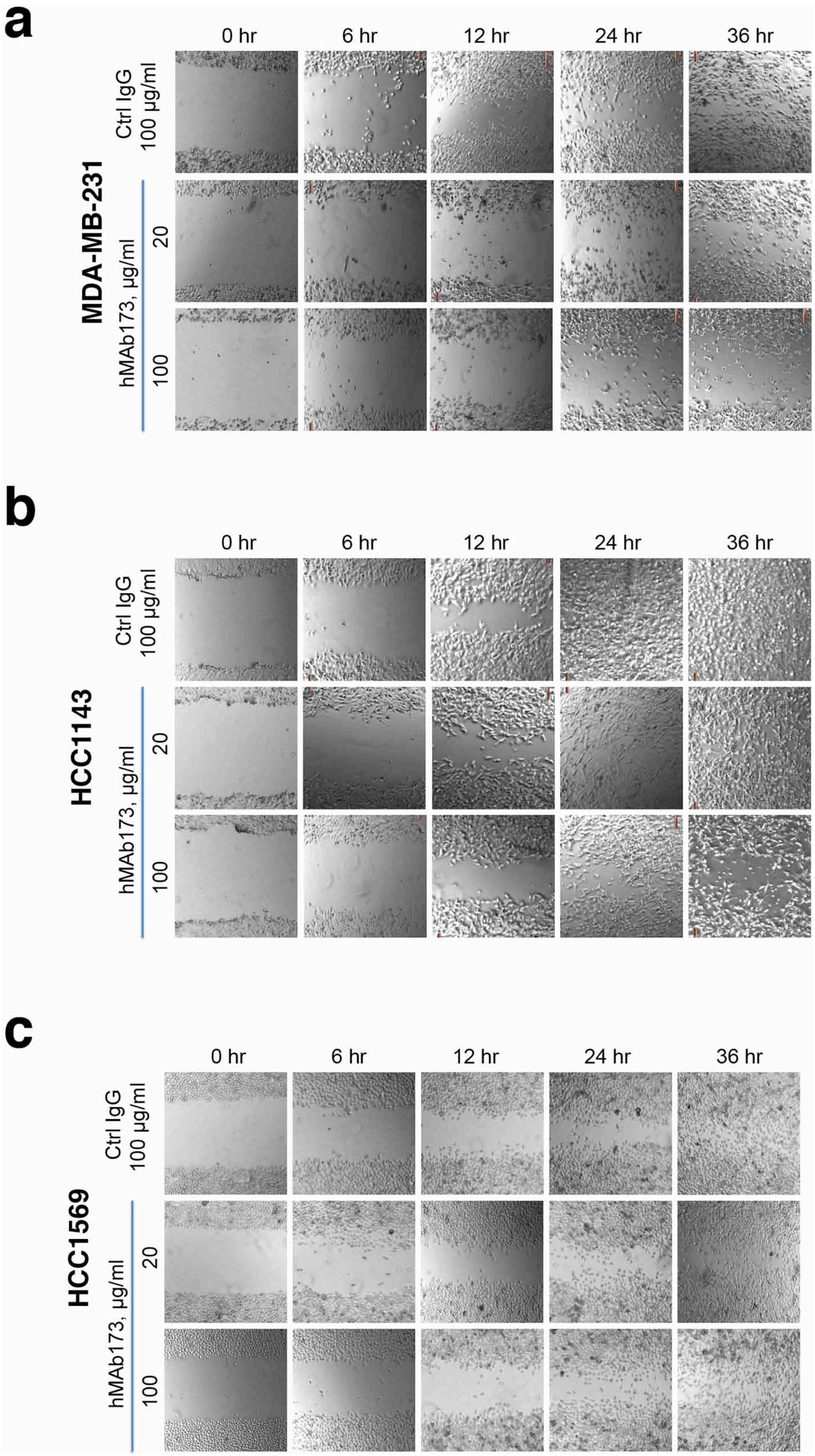

Supplementary Figure S1: Wound-healing assays to measure the cell migration ability of TNBC cells treated with hMAb173. A. MDA-MB-231, **B.** HCC1143 and **C.** HCC1569 cells treated with 20 µg/ml or 100 µg/ml hMAb173. Human IgG (100 µg/ml) was served as the control treatment. Microscopic observations were recorded 0, 6, 12, 24 and 36 hours after scratching the cell surface.

Supplementary Table S1: A list of phosphoPSM identified by phosphotyrosine profiling

Supplementary Table S2: A list of identified phosphotyrosine peptides with normalized relative intensities across the panel of 26 cell lines

Supplementary Table S3: A list of 43 phosphotyrosine peptides differentially phosphorylated between the aggressive and non-aggressive groups

Supplementary Table S4: A list of phosphotyrosine peptides identified in six TNBC cell lines with high expression level of phosphorylated AXL

Supplementary Table S5: AXL IHC staining score and survival data of 57 TNBC patients

Case	SV(months)	Death	AXL Staining
10862	321	0	0
14006	124	0	0
2708	100	0	1
148	94	0	0
4268	88	0	0
6772	84	0	0
11376	78	0	0
12093	76	0	0
982	76	0	0
6741	73	0	0
3809	70	0	0
133	67	0	0
7708	66	0	0
12716	65	0	3
1174	65	0	0
5308	64	0	0
1089	63	0	0
3744	62	0	0
6830	61	0	0
1711	60	0	0
12658	60	0	0
9729	57	0	0
7663	57	0	0
7802	57	0	0
10557	56	0	0
1448	56	0	2
10802	55	0	0
13602	52	0	0
13760	51	0	1
12242	50	0	0
360	49	0	0
10956	47	0	0
4376	47	0	1
6371	46	0	0
7646	46	0	1
7423	44	0	1

(Continued)

Case	SV(months)	Death	AXL Staining
13791	42	1	0
5515	42	0	0
8128	42	0	1
9363	41	0	0
10771	41	0	1
11465	39	0	0
3580	37	1	1
2858	33	1	2
6750	32	1	1
11105	31	1	0
3804	18	1	0
9280	18	1	0
11673	18	1	1
9471	16	1	2
776	14	1	0
4048	14	1	0
4076	13	1	0
4275	13	1	1
10156	9	1	0
7725	6	1	1
2221	6	1	1

Wu et al, 2015 Global phosphotyrosine survey in triple negative breast cancer reveals activation of multiple tyrosine kinase signaling pathways
